# Design and baseline characteristics of the 10 Small Steps Study: a randomised controlled trial of an intervention to promote healthy behaviour using a lifestyle score and personalised feedback

**DOI:** 10.1186/1471-2458-12-179

**Published:** 2012-03-12

**Authors:** Sanjoti Parekh, Corneel Vandelanotte, David King, Frances M Boyle

**Affiliations:** 1 School of Population Health, The University of Queensland, Herston Road, Herston, Queensland 4006, Australia; 2 Healthy Communities Research Centre, The University of Queensland, Salisbury Road, Ipswich, Queensland 4305, Australia; 3 Central Queensland University, Institute for Health and Social Science Research, Bruce Highway, Rockhampton, Queensland 4700, Australia; 4 School of Medicine, The University of Queensland, Herston Road, Herston, Queensland 4006, Australia; 5 Health Systems and Policy Academic Discipline Group, School of Population Health, The University of Queensland, Herston Road, Herston, Queensland 4006, Australia

**Keywords:** Lifestyle score, Non-communicable diseases, Prevalence, Prevention, General practice, Health behaviours, Intervention

## Abstract

**Background:**

Non-communicable diseases (NCDs) are the leading causes of death globally and are associated with a limited set of common, modifiable health behaviours: tobacco use, physical inactivity, harmful use of alcohol and unhealthy diet. General practice offers an ideal avenue for addressing such health behaviours on a population-wide basis. This paper describes the protocol of a multiple health behaviour change intervention designed for implementation in general practice and summarises the baseline characteristics of its participants.

**Method/Design:**

The 10 Small Steps (10SS) study, a randomised controlled trial, involved 4,678 adult general practice patients in Queensland, Australia. Self-reported data were collected to establish the proportion of participants meeting recommended guidelines for ten health behaviours: physical activity, body mass index, alcohol, smoking and six dietary behaviours. Participants were randomised to four groups: contact at baseline only ('single intervention' and corresponding control group) and contact at baseline and 3 months ('dual intervention' and corresponding control group). At each contact the participants received a computer-tailored feedback and one page information sheet according to their allocation to intervention or control groups. Change in the intervention group compared to the control group was assessed at 3 and12 months after baseline data collection.

Responses were summed to calculate an individual lifestyle score (the Prudence Score), which ranged from 0 to 10. The baseline response was 56.5% (4678 of 8343 invited participants) and the study sample was primarily female (68.7%) with an average age of 47 years. The mean Prudence Score was 5.8 (95%CI 5.75-5.85).

**Discussion:**

Baseline data from the 10SS study show that nearly all participants engage in some health behaviours but relatively few adhere simultaneously to a core set of dietary and lifestyle behaviours associated with risk of NCDs. Ample scope exists to improve health behaviour to reduce NCDs in the general practice setting and the 10SS study trial will provide data on the extent to which a minimal computer-tailored intervention can meet this objective. The protocol developed for the 10SS study has potential for translation into routine general practice as it has minimal impact on practice routine whilst contributing to primary prevention objectives.

**Trial Registration:**

The Australian New Zealand Clinical Trials Registry ACTRN12611001213932

## Background

Non-communicable diseases (NCDs) are a major cause of disability and reduced quality of life. The cost of preventable chronic conditions on health care resources and loss of workplace productivity is escalating. Over the last decade there has been a worldwide increased focus on prevention of NCDs [1,2]. Chronic conditions such as cardiovascular disease, type-2 diabetes, obesity and several cancers are associated to varying degrees with a limited set of common, modifiable health behaviours [3-6]. Globally at least 4.9 million people die each year as a result of tobacco use, 2.6 million as a result of being overweight or obese, 4.4 million as a result of raised total cholesterol levels and 7.1 million as a result of raised blood pressure [7]. The total burden of disease attributed to modifiable risk factors such as high blood pressure (7.6%), tobacco (7.8%), alcohol (2.3%), high cholesterol (6.2%), overweight (7.5%), low fruit and vegetable intake (2.1%) and physical inactivity (6.6%) is high [8]. Adoption of a healthy lifestyle has the potential to significantly reduce morbidity and mortality [9-13].

Most primary health care in Australia is provided within general practice clinics and 86% of the population visit a general practitioner (GP) each year [14]. GPs have substantial knowledge of population health and are in an ideal position to advise individual patients about lifestyle choices. Therefore the general practice setting offers the potential to facilitate healthy changes in lifestyle on a large scale [15,16]. Behavioural approaches to both single and multiple risk factors have been applied in general practice and the evidence suggests that brief interventions can be effective [17,18]. Multiple risk factor interventions have been demonstrated to improve health especially in high risk patients. However, there are known barriers to addressing behavioural risk factors in primary care. These include low self-efficacy of GPs for providing health behaviour change information, competing pressures on time, lack of supportive organisational infrastructure or funding to support assessments [19,20]. Overall, the available evidence indicates the need for lifestyle risk factor interventions that can be incorporated into routine care with minimal disruption to general practice activities and limited demands on GPs' time [21,22].

The primary aim of this paper is to describe the protocol for the 10SS study, which was designed to evaluate the use of personalised computer-generated health promotion advice to address multiple health behaviours in the general practice setting. The secondary aim is to describe baseline characteristics of the study participants, including the prevalence and patterns of multiple risk behaviours and their socio-demographic correlates.

## Methods/Design

### Study design and sampling

The 10SS study design was a 2 × 2 randomised controlled trial. The study protocol was designed to answer the specific question: '*Can a low intensity computer-tailored intervention implemented through general practice be used to motivate large numbers of individuals to adopt and maintain a healthier lifestyle and diet?' *There were specifically two parts to this study aim: (i) develop and test an intervention designed to produce short-term (at 3 months after baseline) improvement in ten health behaviours; and (ii) test if a second intervention contact at 3 months improves maintenance of health behaviour change over and above a single one-off intervention at 12 months.

Thirty general practitioners, from metropolitan area in Brisbane, Australia, were invited to participate in the study in 2008. In each participating general practice the practice manager generated a list of potential participants - patients aged between 18 and 70 years who had visited the practice in the preceding six months and had no apparent active cancer, ongoing need for dialysis, recent cardiovascular event, dementia, other terminal illness or recent bereavement. After the treating GP had vetted the list of potential participants to confirm eligibility each patient received a written invitation to take part in the study together with a reply-paid questionnaire. All letters to patients used the corresponding GP's letterhead and included the doctor's electronic signature.

To increase participation in the study non-respondents were sent up to two reminder letters and a new copy of the questionnaire at intervals of three weeks. Completion and return of the questionnaire was regarded as consent to participate in the project. Patients that declined to participate at any stage were subsequently excluded from the study. The data were collected between August and October 2008. Ethics approval for the study was granted by the Behavioural and Social Sciences Ethical Review Committee of the University of Queensland.

### Sample size calculations

The sample size for recruiting the participants was calculated based on the results of the pilot study which is described elsewhere [23]. Using two-sided α (0.05), to have a 95% chance of seeing the proportion scoring 6 or more increase to 45% required 1,756 participants in each group (3,512 overall). A total of 4,220 participants were required to allow for loss to follow-up, and the study therefore aimed to invite 330 patients from each of 20 general practitioners.

### Randomisation

Using each GP as a unit for randomisation, patients who responded at baseline were randomised using a permuted block randomisation procedure. Participants living at the same residential address were randomised into the same group. Study participants were randomised into intervention and control groups and were further randomised into four groups: receiving the intervention at baseline only ('single intervention') and corresponding control group; and receiving the intervention at baseline and 3 months ('dual intervention') and corresponding control group. Participants in the 'single intervention' had study measures assessed at baseline and 12 months, those in the 'dual intervention' were assessed at baseline, 3 and 12 months.

### Intervention

Participants in the intervention group received individualised feedback regarding their lifestyle score described in *Measures *section), and additional health promotion material on behaviours for which they failed to meet guideline recommendations, either once ('single intervention') or twice ('dual intervention'). The health promotion material consisted of concise printed information and links to electronic sites for more extensive information and support. The feedback letter encouraged the adoption of at least one additional health behaviour to those already being adhered to.

### Control

The control group received information about health protective behaviours not included in the Prudence Score (sun protection, updating tetanus vaccination, mammogram and Pap smear). Feedback to the control group was provided in an attempt to reduce attrition.

### Measures

Data were collected from patients via a self-administered questionnaire. This questionnaire was previously piloted in two general practices in Brisbane and was found to be valid (as judged by independent corroboration by an individual's spouse or partner) and reliable (test-retest over an interval of three months) [23]. The questionnaire included 26 items related to modifiable health behaviours and eight related to participants demographics. Demographics included age, gender, education, height, weight, postcode, work status and marital status. The dietary factors assessed included consumption of fish, meat, fruits and vegetables (F&V), milk, spread and salt. Data were collected on current and past smoking status using the established questions from the Australian National Health Survey. The number of alcoholic drinks consumed each day of the week was collected to calculate total alcohol intake. Physical activity questions were taken from the International Physical Activity Questionnaire (IPAQ - Short Form), a valid and reliable measure [24]. Body Mass Index (BMI) (kilograms/meter^2^) was calculated using respondents' self-reported height and weight.

Guidelines promulgated by the National Health and Medical Research Council of Australia (NHMRC) and the National Heart Foundation of Australia (NHF) were used to determine achievement of behavioural recommendations. The guidelines recommend intake of no more than four serves of meat per week [25]; consumption of at least one serve of fish per week [26]; use of reduced or low fat milk [26]; adding no salt during or after cooking [27]; intake of at least five serves of vegetables and two serves of fruit per day [25]; using margarine or other spreads instead of butter [26]; undertaking at least 150 minutes physical activity per week [28]; consumption of two or less standard drinks of alcohol per day with no binge drinking [29]; being a non-smoker [30] and having body mass index between 18.49 and 24.99 kg/m^2 ^[31].

Responses to the ten behavioural items were dichotomized as meeting (1 point) or not meeting (0 point) the criteria and summed to produce a composite lifestyle score, the Prudence Score. The score ranged between 0 and 10. The Prudence Score was used as the main outcome variable. Independent variables were gender (female/male); age group (18 to 39 yrs/40 to 59 yrs/60 and above); marital status (married or living as married/single, divorced, widowed, never married); employment status (working full or part time/not working due to health, retirement, home duties) and education (less than high school/high school/diploma or trade certificate/university).

Socio-economic Index for Areas (SEIFA) categories were applied as an area level indicator of socioeconomic status and were based on the postcodes provided for each patient. SEIFA ranks areas according to socio-economic and positional disadvantage based on information derived from the five-yearly Census of Population and Housing in Australia, [32] and is the most widely used general measure of socio-economic status (SES) by area in Australia. The SEIFA sub-code for 'Economic Resources', which includes variables such as income, housing expenditure and assets of households, was used for this analysis. Ten categories of SEIFA were merged into three categories: advantaged, moderately disadvantaged and disadvantaged.

## Data management and statistical analysis

After data entry, data was manually and statistically checked as a part of the data-cleaning process. Initially, descriptive statistics were calculated for all variables. To identify patterns of health behaviours in the study population, the Prudence Score was stratified by demographic characteristics of gender, age, education, marital and employment status. A bivariate analysis using the Chi2 test was performed to assess differences between meeting health behaviour recomm and the demographic variables of gender, education, work status, marital status, and area of residence. A two-sample *t*-test was used to assess the differences in the mean Prudence Score for gender, age, education, employment status, marital status and SEIFA.

The Prudence Score was further divided into 3 categories: low (0 to 5), medium (6 & 7) and high (8 to 10) as the primary outcome variable in the multivariate analysis. The multivariate logistic model was used to further assess the impact of each demographic factor on the combined score as well as determine characteristics of individuals at highest risk of having unhealthy lifestyles. Results of this model are reported as relative risk ratios. Records for missing values for any of the prudent variables were deleted from the total Prudence Score calculations. The statistical package STATA version 11 (Stata Corporation, 2008) was used for the analysis.

### Response rate

**Figure 1:**
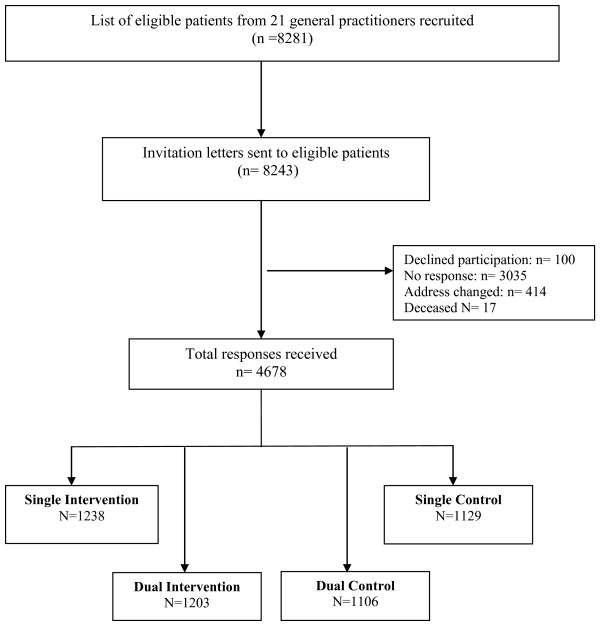
**Flow diagram for the study**.

Of the 30 GPs approached, 25 showed initial interest. Four GPs subsequently withdrew because of leave planned during the recruitment period or inadequate computer systems to identify potentially eligible patients. Practice staff identified a total of 8,281 (32% men) potentially eligible patients for the remaining 21 GPs, of whom 4,678 completed and returned the questionnaire. As such, the participation rate at baseline was 59.9%, after notified deaths and returns to sender were omitted (5.2%; n = 412). Figure 1 presents a study flow chart.

### Baseline characteristics of the sample

Baseline socio-demographic characteristics for intervention and control groups are presented in Table 1. The average age of participating patients was 47 years and the majority were female (68.7%), married or living as married (68.8%), and with a diploma or university degree (56.6%).

**Table 1:** Socio-demographic and health behaviour characteristics at baseline [mean(± SE) and number(percentages)]

CHARACTERISTICS	ALL PERSONS	INTERVENTION	CONTROL	P-VALUE
**DEMOGRAPHICS**				

MEAN AGE	49.7(± 1.1)	49.8(± 1.1)	49.7(± 1.2)	0.846

GENDER (% WOMEN)	3229(68.7%)	1688(68.0%)	1541(69.5%)	0.257

MARRIED OR LIVING AS MARRIED	3233(69%)	1690(68.9%)	1543(69.1%)	0.470

EDUCATION (%TERTIARY)	2660(56.8%)	1406(52.9%)	1254(56.2%)	0.202

**HEALTH BEHAVIOURS**				

Meat Intake ≤ 4 Serves Per Week	3245(69.2%)	1689(68.8%)	1556(69.5%)	0.313

Fish Intake ≥ 2 Serves Per Week	3186(67.8%)	1666(67.9%)	1520(67.8%)	0.485

Use Of Low Or No Fat Milk	3254(69.3%)	1688(68.7%)	1566(69.9%)	0.193

Salt: No Added Salt	2033(43.4%)	1056(43.2%)	977(43.6%)	0.397

Vegetables And Fruit: 7 Serves Per Day	579(12.4%)	317(13.0%)	262(11.7%)	0.102

Use Of Spreads Other Than Butter	3109(66.3%)	1639(66.9%)	1470(65.6%)	0.181

Physical Activity≥150 Minutes Per Week	2356(50.3%)	1231(50.2%)	1125(50.4%)	0.469

Alcohol ≤ 2 Standard Drinks Per Day	3216(68.4%)	1671(68.0%)	1545(68.9%)	0.271

No Smoking	4027(86.1%)	2105(86.0%)	1922(86.3%)	0.381

Body weight between 18.5 to 24.99 kg/m2	1861(41.0%)	1003(42.2%)	858(39.8%)	0.147

*T*-Test Tested Continuous Variables

Chi-Squared Tested Categorical Variables

### Distribution of health behaviours

Table 1 summarizes the distribution of the dietary and lifestyle behaviours for all the participants. Most (86%) were non-smokers whereas only 12.4% consumed 7 or more serves of vegetables and fruits daily. These ten behaviours are further stratified by gender and age (Tables not included). Women more soften reported adhering to several behaviours than men: eating 4 or less serves of meat per week (74.6% versus 57.2%, p < 0.05), drinking low fat milk (72.7% versus 61.8%, p < 0.05) and drinking alcohol within recommended limits (74.0% versus 56.6%, p < 0.05). For 46.4% of women BMI was within the recommended range, as compared to 29.6% of men. Older patients reported significant higher adherence to recommended behaviours, except for BMI where older patients had lower adherence. No age specific differences were noted for physical activity and salt intake. A significantly lower percentage of married patients had BMI within normal range when compared to patients who were single, widowed or divorced (40.9% versus 50.5%, p < 0.05).

### Combinations of health behaviours

The data were analyzed to assess the proportion of participants that adhered to certain combinations of multiple health behaviours. Only 30% of patients reported they were non-smokers and adhered to physical activity and alcohol guidelines (see Table 2). This proportion dropped to 5.1% when adding a fourth behaviour, adherence to recommended daily F&V intake. Only 2.8% of the study population adhered to all four behaviours and also had normal body weight (18.49 to 24.99 kg/m^2^).

**Table 2:** Proportion of the general practice population adhering to recommendations for combined health behaviours

	% adherence to health behaviours						
	**N**	**None**	**One**	**Two**	**Three**	**Four**	**All Five**

**Smk**^**a**^	4654	13.9	86.1				

**Smk+Alc**^**b**^	4645	7.5	30.4	62.1			

**Smk+Alc+Pa**^**c**^	4447	3.2	17.6	49.2	30.0		

**Smk+Alc+PA+VF**^**d**^	4437	3.0	16.8	43.7	31.3	5.1	

**Smk+Alc+PA+BMI**^**e**^	4311	1.6	11.6	36.0	36.1	14.7	

**Smk+Alc+PA+BMI+VF**	4303	1.5	11.2	32.4	34.8	17.3	2.8

a: Non-smoker

b: Alcohol two or less standard drinks per day or a non-drinker

c: Following physical activity guidelines (150 minutes of moderate or 60 minutes of vigorous physical activity per week)

d: Greater than or equal to 7 serves of fruit and vegetables per day

e: Body mass index between 18.49 kg/m^2 ^and 24.99 kg/m^2^

### Prudence Score

The mean Prudence Score for the study sample was 5.80 (95% CI 5.75-5.85), with scores approximating a 'normal' distribution. Less than 1% reported a Prudence score of '0', similarly only 2.8% adhered to five important recommendations: BMI within normal range, sufficient physical activity, within limits alcohol intake, being a non-smoker and sufficient F&V intake. Women had a significantly higher age-standardized mean Prudence Score than men (5.98 versus 5.41, t = 10.57; df = 4065; p < 0.001). Table 3 shows the mean Prudence Score was lower in men than in women for all age-groups, but tended to increase with age in both genders. Patients with tertiary education had higher scores than those with high school education (5.98 versus 5.55; t = 8.02, df = 4050, p < 0.001). Regardless of educational background, marital status or employment status, women reported following a healthier lifestyle than men (Table 3).

**Table 3:** Distribution of mean Prudence Score by socio-demographic variables and effect of gender

	Total			Men			Women		
	**Mean**	**95%CI**	P-value*	**Mean**	**95%CI**	P-value*	**Mean**	**95%CI**	P-value*

**All **(N = 4281)	5.80	5.75-5.85							

**Gender**									

Female (N = 2928)	5.98	5.92-6.04							

Male (N = 1353)	5.41	5.32-5.50	< 0.001						

**Age Group (Years)**									

< 50 years (N = 2170)	5.56	5.49-5.63	< 0.001	5.00	4.87-5.14	< 0.001	5.76	5.68-5.84	< 0.001

50 and above (N = 1893)	6.08	6.01-6.15		5.75	5.63-5.87		6.28	6.19-6.36	

**Education Level**									

University/Diploma/trade N = 2366	5.98	5.91-6.04	< 0.001	5.63	5.52-5.75	< 0.001	6.16	6.08-6.23	
					
High School and below N = 1686	5.55	5.47-5.63		5.03	4.89-5.17		5.76	5.67-5.86	

**Marital Status**									

Married (N = 2809)	5.84	5.78-5.90		5.49	5.39-5.60		6.03	5.95-6.10	
					
Other (N = 1252)	5.71	5.62-5.81	0.02	5.16	4.99-5.33	0.001	5.91	5.80-6.01	0.07

**Employment Status**									

Employed (N = 2897)	5.77	5.71-5.83		5.39	5.29-5.49		5.96	5.89-6.03	
					
Not employed (N = 1159)	5.88	5.79-5.98	0.04	5.47	5.28-5.67	0.31	6.03	5.92-6.14	0.33

**Socioeconomic index of economic resources**									

Advantage (N = 1987)	5.95	5.87-6.02		5.58	5.44-5.71		6.10	6.02-6.18	
					
Disadvantaged (N = 2073)	5.67	5.60-5.74	< 0.001	5.27	5.15-5.39	< 0.001	5.87	5.79-5.96	< 0.001

*Two sample *T*-test used for assessing the difference between sub-groups

Table 4 displays the results of a multinomial regression model examining associations between socio-demographic factors and 3 categories of the Prudence Score. After simultaneous adjustment for socioeconomic status and all other factors in the model there was an increased risk of unhealthy diet and lifestyle for males (Relative Risk Ratio (RRR) = 3.03; 95% CI = [2.42-3.79]), younger age (RRR = 3.76; [2.83-5.01]) and lower educational attainment (RRR = 2.82; [2.11-3.76]). Of these factors, age between 18 to 39 years and educational attainment below high school were the strongest predictors of unhealthier lifestyle behaviours. As the interaction terms were significant for age and gender (*χ*^2 ^= 180.4, df = 10, p < 0.05) as well as for education and employment status (*χ*^2 ^= 83.0, df = 14, p < 0.05), the main effects model was adjusted for these potential confounders. Following the re-analysis marital status and employment status were no longer significantly associated with Prudence Score.

**Table 4:** Multivariate associations between Prudence Score and socio-demographic variables

Prudence Score*			
	**Low Score: High Score**	**Medium Score: High Score**	**Test of Significance^#^**

**Gender**			

Female	1	1	

Male	3.03 (2.42-3.79)	1.69(1.35-2.11)	P < 0.001

**Age group**			

60+	1	1	
	
40 to 59	2.32 (1.80-2.99)	1.52 (1.20-1.93)	P < 0.001
	
18 to 39	3.76 (2.83-5.01)	1.63 (1.24-2.15)	

**Marital Status**			

Married	1	1	P = 0.18
	
Other	1.19 (0.97-1.47)	1.07 (0.87-1.31)	

**Employment Status**			

Employed	1	1	
	
Not Employed	1.00 (0.80-1.25)	0.85(0.69-1.06)	P = 0.12

**Education**			

University	1	1	
	
Diploma or trade	1.47 (1.15-1.87)	1.15 (0.91-1.45)	
	
High school	1.96 (1.50-2.55)	1.31 (1.02-1.70)	P < 0.001
	
Less than high school	2.82(2.11-3.76)	1.52 (1.14-2.01)	

**Socioeconomic Status index for economic resources**			

Advantaged	1	1	
	
Disadvantaged	0.91 (0.71-1.18)	0.96 (0.76-1.23)	P = 0.22

Most disadvantaged	0.81 (0.55-1.18)	0.92 (0.63-1.34)	

*Prudence Score defined as low (0 to 5), medium (6 & 7) and high (8 to 10)

#Adjusted Wald tests of significance of individual parameters after adjustment for other variables in the model.

## Discussion

This paper describes the study procedures for the 10 Small Steps (10SS) study and provides the baseline characteristics of a sample of the 4678 participants who took part in the study. The 10SS approach differs from other studies in its focus by delivering a health behaviour intervention with minimal interruption to general practice routine care and communicating health behaviour advice using a lifestyle score. The Prudence Score showed that despite a relatively high adherence to at least some health behaviours, there was considerable scope for improvement. Only 2.8% of general practice patients adhered to five important protective behaviours: being a non-smoker, sufficient physical activity, safe alcohol intake, eating 7 or more serves of fruit and vegetables daily, and having a normal BMI. Providing computer-tailored feedback that was endorsed by the patient's GP might have contributed to the participation of a relatively large cohort. This cohort will provide the power to continue with a factorial randomised trial with a good chance of detecting even small changes in behaviour attributable to this minimal intervention.

The dietary and lifestyle items included in the study are based on epidemiological evidence demonstrating their association with morbidity and mortality [11,29,33-36]. Behaviours such as smoking and physical activity have direct evidence for prevention of NCDs, whereas other behaviours, such as drinking low fat milk and using spreads other than butter, are indirectly supported due to their role in reducing daily saturated fat intake. A previous study by Spencer and Jamrozik et al., using eight of the items used in this study, suggested that in a large cohort of elderly men the combined score had a linear association with all-cause mortality over the subsequent five years and each health behaviour was individually associated with reduced risk of death due to Myocardial Infarction [13,37].

While the protocol developed for this study proved feasible in a clear majority of practices that were approached, the uptake of invitations by patients was incomplete and the predominance of female participants reflected the low 'visibility' of men attending health services, especially in the first half of adult life. Australian data report that female patients account for 57.6% of General Practice consultations [14]. The consistently higher Prudence Score among older participants and women is notable, and consistent with previous reports that conclude women to be more health conscious than men [38,39]. Another explanation could be that females and older participants have more frequent health service contacts, each of which provides an occasion for opportunistic health promotion advice [39] Our data suggest that alternative ways to engage men in health promotion activities need to be sought. For example, the addition of a lifestyle score during a 'Men's Health check' (for Australian men between 45-49 years), might be helpful to increase participation of men in health promotion activities.

The main limitation on this study is the non-response from 40% of invited patients; however, this response fraction is similar to that of other large community surveys [40,41]. Furthermore, the data collected on diet and lifestyle is comparable with other population-wide data for Australia. For example, 86.1% of participants were non-smokers (never- and ex-smokers combined) compared with a national figure of 83.4% [42]. The mean BMI was 26.7 kg/m2, with 56.1% of participants in the 'overweight' or 'obese' range, which is again consistent with a national estimate of 58.5% [43]. Despite the underrepresentation of males, the study sample may be considered broadly representative of the adult Australian population.

A strength of this protocol is the systematic approach to data collection. The data collection tool is a reliable and valid instrument [23] that can be used in clinical settings to examine and measure multiple risk behaviours associated with chronic conditions. The added value of the Prudence Score is its ease of application without requiring invasive biological measures. It will help to identify patients at higher risk of non-communicable diseases, as well as guide the development of effective strategies for risk-reduction and prevention. The protocol was successfully implemented in order to collect the data and to test the intervention. This study protocol may be useful in general practice for measuring multiple health behaviours simultaneously and communicating the results to patients using the Prudence Score. The next step in the 10SS study is to evaluate the extent to which this minimal computer-tailored intervention leads to positive health behaviour change in the short (three months) and long term (12 months).

## Abbreviations

10SS: 10 Small steps; NCDs: Non-communicable diseases; BMI: Body mass index; GPs: General practitioners; NHMRC: National health and medical research council; NHF: National heart foundation of Australia; SES: Socio-economic status.

## Competing interests

The authors declare that they have no competing interests.

## Authors' contributions

SP made a substantial contribution to the concept, design, data collection, data management, data interpretation and drafting the manuscript. CV supervised development of the computer-tailored health promotion advice as well as contributed to the draft. DK contributed to the development of the study, data interpretation and was involved in drafting the manuscript. FB supervised the implementation of the study and was involved in drafting the manuscript. All authors read and approved the final manuscript.

## Pre-publication history

The pre-publication history for this paper can be accessed here:

http://www.biomedcentral.com/1471-2458/12/179/prepub

## Supplementary Material

Additional file 1 **Promoting Healthy Communities**. Click here for file
