# Supplementary material for: Design and baseline characteristics of the 10 Small Steps Study: a randomised controlled trial of an intervention to promote healthy behaviour using a lifestyle score and personalised feedback
Source: BMC Public Health. 2012 Mar 12;12:179. doi: 10.1186/1471-2458-12-179 (PMC3328259; doi:10.1186/1471-2458-12-179)
Supplement: Additional file 1 — Promoting Healthy Communities. [file 1471-2458-12-179-S1.DOC]

**Promoting Healthy Communities**

**Instructions**

- Please answer by circling the appropriate number or by writing your answer in the space provided.
- Please check that you have answered all the questions before posting this survey back to us.
- The far right hand column of each page is for **Office use only**.

|  | **EATING HABITS** | **Office Use Only** |
| --- | --- | --- |
|  |  |  |
| Q. 1 | How many times do you eat red meat in an average week, including sausages, luncheon meat, salamis, meat pies, hamburger or bacon (but not including fish or poultry)? (*Please circle ONE number*) |  |
|  |  |  |
|  | 1. Ten or more times per week |  |
|  | 2. Five to nine times a week |  |
|  | 3. Three to four times a week |  |
|  | 4. Once or twice a week |  |
|  | 5. Less than once a week |  |
|  | 6. Never |  |
|  |  |  |
| Q. 2 | How often do you eat fish or seafood in an average week? |  |
|  |  |  |
|  | 1. Six or more times a week |  |
|  | 2. Three to five times a week |  |
|  | 3. Once or twice a week |  |
|  | 4. Less than once a week |  |
|  | 5. I never eat fish for medical reasons |  |
|  | 6. I never eat fish for religious or ethical reasons |  |
|  | 7. I never eat fish for other reasons (Please specify)_________ |  |
|  |  |  |
| Q. 3 | What type of milk or milk products do you **usually** drink or use in tea or coffee? |  |
|  |  |  |
|  | 1. Condensed |  |
|  | 2. Full cream (normal milk) |  |
|  | 3. Almost equal amounts of full cream and reduced fat |  |
|  | 4. Reduced fat |  |
|  | 5. Skim |  |
|  | 6. None |  |
|  | 7. Other (Please specify) ___________________ |  |
|  |  |  |
| Q. 4 | How often do you add salt to your food during or after it is cooked? |  |
|  | 1. Never |  |
|  | 2. Rarely |  |
|  | 3. Sometimes |  |
|  | 4. Almost always |  |
|  | 5. Always |  |
|  |  |  |
| Q. 5 | How many servings of vegetables do you eat each day?  (1 serving=1/2 cup of cooked vegetables or 1 cup of salad) |  |
|  |  |  |
|  | 1. Less than one |  |
|  | 2. One per day |  |
|  | 3. Two per day |  |
|  | 4. Three per day |  |
|  | 5. Four per day |  |
|  | 6. Five per day |  |
|  | 7. Six per day |  |
|  | 8. Seven or more per day |  |
|  |  |  |
| Q .6 | How many servings of fruit do you eat each day?  (1 serving =1 large fruit like an apple or banana or 8 strawberries or 20 grapes or 20 cherries or 2 smaller fruits such as kiwi fruit, apricots, plums or ½ cup of juice or 4 dried apricots or 1½ tablespoon of sultanas or 1 cup of canned fruit ) |  |
|  |  |  |
|  | 1. Less than one serve per day |  |
|  | 2. One serve per day |  |
|  | 3. Two serves per day |  |
|  | 4. Three serves per day |  |
|  | 5. Four or more serves per day |  |
|  |  |  |
| Q. 7 | What kind of spread do you usually use with bread, toast, muffins, crumpets etc? |  |
|  |  |  |
|  | 1. Butter |  |
|  | 2. Margarine |  |
|  | 3. None |  |
|  | 4. Other (Please specify) _________________ |  |
|  |  |  |
|  |  |  |
|  |  |  |
|  | **RECREATION, SPORT AND FITNESS** |  |
|  |  |  |
|  | The section on the next page asks about the time you spent **walking** in the **past 7 days.** This includes walking at work and at home, walking to travel from place to place and any other walking that you did solely for recreation, sport, exercise or leisure. |  |
| Q. 8 | During the last 7 days, on how many days did you walk for at least 10 minutes at a time? |  |
|  | 1. ____________ days 2. None (Go to Q. 11 ) |  |
|  |  |  |
| Q. 9 | Please estimate how much time in total did you usually spend walking on one of those days? |  |
|  | **_________ / ___________**  Hours / minutes |  |
|  |  |  |
| Q.10 | When you are walking, at what pace do you usually walk? |  |
|  |  |  |
|  | 1. Slow pace |  |
|  | 2. Intermediate pace (medium) |  |
|  | 3. Fast pace (Briskly) |  |
|  |  |  |
|  | In this section we would like to find out about all the **moderate** **physical** **activities** that you did in last 7 days. This refers to activities that take moderate physical effort and make you breathe somewhat harder than normal. Think only about those physical activities that you did for **at least 10 minutes at a time**. |  |
|  |  |  |
| Q. 11 | During the last 7 days, on how many days did you do moderate physical activities?  *(eg carrying light loads, bicycling at a regular pace, or doubled tennis)* Do not include walking. |  |
|  | 1. _________ days 2. None (Go to Q. 13) |  |
|  |  |  |
|  |  |  |
| Q. 12 | Please estimate how much time in total did you usually spend on one of those days doing moderate physical activities? |  |
|  | **_________ / ___________**  Hours / minutes |  |
|  |  |  |
|  |  |  |
|  | This section asks about any **vigorous** **activity** you do in a **usual week at least for 10 minutes at a time**. Vigorous physical activities refer to activities that take hard physical effort and make you breathe much harder than normal. Think only about those physical activities that you did for at least 10 minutes at a time. |  |
|  |  |  |
| Q. 13 | During the last 7 days, on how many days did you do **vigorous** **physical activities** like heavy lifting, digging, aerobics, or fast bicycling? |  |
|  |  |  |
|  | 1. _________ days 2. None (Go to Q. 15) |  |
|  |  |  |
|  |  |  |
| Q. 14 | Please estimate how much time in total did you usually spend on one of those days doing vigorous physical activities? |  |
|  |  |  |
|  | **_________ / _______** Hours/ minutes |  |
|  |  |  |
|  | **SMOKING** |  |
|  |  |  |
|  |  |  |
| Q. 15 | How often do you currently smoke cigarettes, cigars, pipes or other tobacco products? |  |
|  |  |  |
|  | 1. Daily |  |
|  | 2. At least weekly (Not Daily) |  |
|  | 3. Less often than weekly (Go to Q. 17) |  |
|  | 4. Not at all (Go to Q. 17) |  |
|  |  |  |
|  |  |  |
| Q. 16 | On average, how many cigarettes do you smoke per day or each week now? |  |
|  |  |  |
|  | ------------------ cigarettes per day **OR** |  |
|  | ------------------ cigarettes each week **OR** |  |
|  | ------------------ grams of tobacco per week |  |
|  |  |  |
|  |  |  |
| Q. 17 | Over your lifetime, have you smoked at least 100 cigarettes? |  |
|  |  |  |
|  | 1. YES (Go to Q. 18) 2. NO (Go to Q. 19) |  |
|  |  |  |
| Q. 18 | Have you ever smoked tobacco daily? |  |
|  |  |  |
|  | 1. Yes, I smoke daily now. |  |
|  | 2. Yes, I used to smoke daily. |  |
|  | **⮡** When did you finally stop smoking daily? |  |
|  | Day________ of __________month of ________ year  **OR** ____________ weeks ago or  ____________ months ago or  ____________ years ago  **OR** when I was _________ years old |  |
|  | 3. No, I have never smoked daily. |  |
|  |  |  |
|  |  |  |
|  |  |  |
|  |  |  |
|  | **ALCOHOL** |  |
|  |  |  |
| Q. 19 | Have you drunk alcohol in the last year? |  |
|  |  |  |
|  | 1. YES (Go to Q. 20) |  |
|  | 2. NO (Go to Q. 21) |  |
|  |  |  |
| Q. 20 | **IF YES**, how many “standard” drinks do you have each day in a **usual** week?  (**1 “standard” drink** = 1 middy of full-strength (5%) *beer*, or 1 pub measure of *spirits*, or 1 glass of *sherry or port*, or 1 glass of *wine* or 1.5 middies of XXXX *Gold* or 2 middies of *Toohey’s* 2.2 or 5 middies of *Toohey’s Light*) |  |
|  |  |  |
|  | ____________ drinks on Monday |  |
|  | ____________ drinks on Tuesday |  |
|  | ____________ drinks on Wednesday |  |
|  | ____________ drinks on Thursday |  |
|  | ____________ drinks on Friday |  |
|  | ____________ drinks on Saturday |  |
|  | ____________ drinks on Sunday |  |
|  |  |  |
|  |  |  |
|  | **OTHER HEALTH-RELATED INFORMATION** |  |
|  |  |  |
| Q. 21 | When did you last have an injection to protect against tetanus? |  |
|  |  |  |
|  | 1. At the age of __________ years **OR** |  |
|  | 2. ________________ years ago **OR** |  |
|  | 3. I do not remember when I last had a tetanus needle |  |
|  | 4. Never |  |
|  |  |  |
| Q. 22 | How often do you use sunscreen when you go out in the sun for 10 minutes or more between 10am and 3 pm?  (*Please circle ONE number*) |  |
|  |  |  |
|  | 1. Always |  |
|  | 2. More than half the time |  |
|  | 3. About half the time |  |
|  | 4. Less than half the time |  |
|  | 5. Never |  |
|  |  |  |
|  |  |  |
| Q. 23 | How often do you wear a hat that shades your face, neck and ears when you go out in the sun for 10 minutes or more between 10am and 3pm? |  |
|  |  |  |
|  | 1. Always |  |
|  | 2. More than half the time |  |
|  | 3. About half the time |  |
|  | 4. Less than half the time |  |
|  | 5. Never |  |
|  |  |  |
| Q. 24 | How often do you wear a full sleeve shirt to cover your body when you go out in the sun for 10 minutes or more between 10am and 3pm? |  |
|  |  |  |
|  | 1. Always |  |
|  | 2. More than half the time |  |
|  | 3. About half the time |  |
|  | 4. Less than half the time |  |
|  | 5. Never |  |
|  |  |  |
| Q. 25 | **This question is for women only.** |  |
|  | When did you last have a Pap Smear test or cervical screening test? |  |
|  |  |  |
|  | 1. Less than 2 years ago |  |
|  | 2. More than 2 years ago |  |
|  | 3. I have never had this test |  |
|  | 4. I have had a Pap Smear in the past but cannot remember when |  |
|  | 5. I no longer need to have Pap smears because of surgery I have had in the past |  |
|  |  |  |
| Q. 26 | **This question is only for women over the age of 50 years.** |  |
|  | When did you last have a mammogram? |  |
|  |  |  |
|  | 1. Less than 2 years ago |  |
|  | 2. More than 2 years ago |  |
|  | 3. I have never had a mammogram |  |
|  | 4. I have had a mammogram in the past but cannot remember when |  |
|  |  |  |
|  | **HEIGHT AND WEIGHT** |  |
|  |  |  |
| Q. 27 | How tall are you without shoes? |  |
|  |  |  |
|  | ___________ centimetres |  |
|  |  |  |
|  | OR _________feet ___________inches |  |
|  |  |  |
|  |  |  |
|  |  |  |
| Q. 28 | How much do you weigh without clothes and shoes? |  |
|  |  |  |
|  | ___________ kilograms |  |
|  |  |  |
|  | ___________ stone _________ pounds |  |
|  |  |  |
|  |  |  |
|  | **BACKGROUND INFORMATION** |  |
|  |  |  |
| Q. 29 | How old are you now? |  |
|  | _________ years |  |
|  |  |  |
| Q. 30 | What rules do you have about smoking in your home? |  |
|  |  |  |
|  | 1. We do not permit smoking anywhere inside or outside the home |  |
|  | 2. We only allow smoking outside the home |  |
|  | 3. We allow smoking only in certain rooms |  |
|  | 4. We do not have any rules about smoking in or around the home |  |
|  |  |  |
| Q. 31 | What is your current marital status? |  |
|  |  |  |
|  | 1. Married or living as married |  |
|  | 2. Separated |  |
|  | 3. Widowed |  |
|  | 4. Divorced |  |
|  | 5. Never Married |  |
|  |  |  |
| Q 32 | Please circle one answer to each of the following statements |  |
|  |  |  |
|  | 1. I am already on a special diet.  1. Yes 2. No |  |
|  | 2. I have had an injury or illness that prevents me from exercising at present.  1. Yes 2. No |  |
|  | 3. I am pregnant which affects my diet and lifestyle  1. Yes 2. No |  |
|  | 4. I have a long-term medical problem or allergies that affect my diet and lifestyle  1. Yes 2. No  If Yes, please specify the problem or food to which you are allergic  ------------------------------------------------------------------------------------------------------------------------------------------------------------------ |  |
|  |  |  |
| Q 33 | Which of the following describes you at present? |  |
|  |  |  |
|  | 1. Working full-time |  |
|  | 2. Working part-time |  |
|  | 3. Not working (Go to Q 35) |  |
|  | 4. Retired due to age (Go to Q 35) |  |
|  | 5. Retired due to health (Go to Q 35) |  |
|  | 6. Student |  |
|  | 7. home duties |  |
|  |  |  |
| Q 34 | If you have a paid job at present |  |
|  | 1. What is the title of the job?  Please specify ____________________________ |  |
|  | 2. Please describe the main activities of the job  ________________________________________________  _________________________________________________ |  |
|  | 3. Is most of your day at work spent sitting?  1. Yes 2. No |  |
|  | 4. Is most of your day at work spent standing at one place?  1. Yes 2. No |  |
|  |  |  |
| Q 35 | What is the highest level of education you have completed? |  |
|  |  |  |
|  | 1. University degree |  |
|  | 2. Diploma or trade certificate |  |
|  | 3. High school (Year 12 or equivalent) |  |
|  | 4. Some high school |  |
|  | 5. Primary school |  |
|  | 6. No formal education |  |

***Thank you for completing this form.***

**Please, make sure you have answered all the questions.**

**Please, send the form back to us in the envelope provided.**
